# Allergen specific sublingual immunotherapy (ASSIT) reduces Il-4 and enhances interferon-gamma intracellular expression by Cd8+ T-cells in perenneal allergic rhinitis (Par)

**DOI:** 10.1186/1710-1492-8-S1-A25

**Published:** 2012-11-02

**Authors:** Farag I Farag – Mahmod, Wahid AM Ali, Waheed F Haysam, Sahar Z Zakaria, Gehan Elhadidy

**Affiliations:** 1 Allergy/Immunology Unit, Faculty of medicine, Suez Canal University, Ismailia, Egypt

## Background

ASSIT is a novel therapy of allergic diseases that gained significant acceptance over the past decade. This study was undertaken to investigate whether ASSIT in PAR may influence the Intracellular expression of IFN- gamma and IL-4 by CD3+CD8+ T-cells.

## Subjects and methods

Twenty adult PAR patients sensitive only to the mite D.farinae as diagnosed by Prick skin testing (PST) [Omega labs, Montreal, Canada] were included in the study. ASSIT to D. farinae was administered for 6 months.

Flow cytometric evaluation of the intracellular expression of IL-4 and IFN- gamma by CD3+ CD8+ T-cells was determined according to Manufacturer instructions (Beckton Dickenson) before and after ASSIT.

The total 5 symptom score (T5SS) of PAR and the diameter of PST were also examined.

## Results

After ASSIT; the percentage of Il-4 expressing CD8+ T-cells significantly decreased from [0.69 +/- 0.18] to [0.33 +/- 0.13] and the percentage of IFN-gamma expressing CD8+ T-cells significantly increased from [4.63 +/- 1.29] to [7.20 +/- 2.09]. The T5SS and the PST diameter also significantly decreased.

## Conclusion

In this study the favorable clinical response induced by ASSIT in PAR correlated with the decrease in the percentage of Tc2 cells and the increase in the percentage of Tc1 cells.

